# The Intravenous Drug User with a Big Heart

**DOI:** 10.7759/cureus.3325

**Published:** 2018-09-18

**Authors:** Abhishek Bhagat, Frank H Annie, Vallabh Karpe, Alfred Tager, Sarah Nease

**Affiliations:** 1 Internal Medicine, Charleston Area Medical Center, Charleston, USA; 2 Cardiology, Charleston Area Medical Center, Charleston, USA; 3 Emergence Medicine, Charleston Area Medical Center, Charleston, USA

**Keywords:** illicit drug use, endocarditis, infective endocarditis, intravenous drug use

## Abstract

Infective endocarditis is a severe and now more frequently encountered condition given the rise of intravenous (IV) drug use. An IV drug user presented with septic shock and bacterial endocarditis. Upon imaging, a fistulous tract was discovered, communicating from the annulus of the infected mitral valve to a large left ventricular pseudoaneurysm (PA). Presence of valvular vegetation, heart failure, and PA are all independent factors of increased mortality rates. The sheer size of the PA placed this patient at a very high surgical risk, and she was deemed inoperable. She was discharged when stabilized on supportive medical therapy. However, she returned six weeks later in cardiogenic shock with multi-organ failure. Repeat imaging revealed that the PA had significantly increased in size. Despite optimal medical management, the patient’s condition deteriorated, and she, unfortunately, succumbed to her illness.

## Introduction

Infective endocarditis (IE) is a severe and now more frequently encountered condition among intravenous (IV) drug users. The incidence of IE in IV drug users is 2-5% per year, accounting for 5-20% of hospital admissions and responsible for 5-10% of overall mortality [1]. With the rise of IV drug use in recent years, IE related hospitalizations have increased correspondingly [2]. Even though there is often multi-organ system involvement, cardiac complications (perivalvular abscesses, heart blocks, valvular dysfunction, etc.) tend to be the most common type in IE complications. Additionally, accompanying heart failure has shown to have the most significant impact on prognosis [3]. Aneurysms or pseudoaneurysms (PA) of the left ventricle (LV) have traditionally been described as a rare complication of myocardial infarction (MI) or cardiac surgery. However, IE leading to an intracardiac fistula and subsequently resulting in a left ventricular pseudoaneurysm (LVPA) has been very rarely reported. We present an interesting case complicated by such findings.

## Case presentation

**Figure 1:**
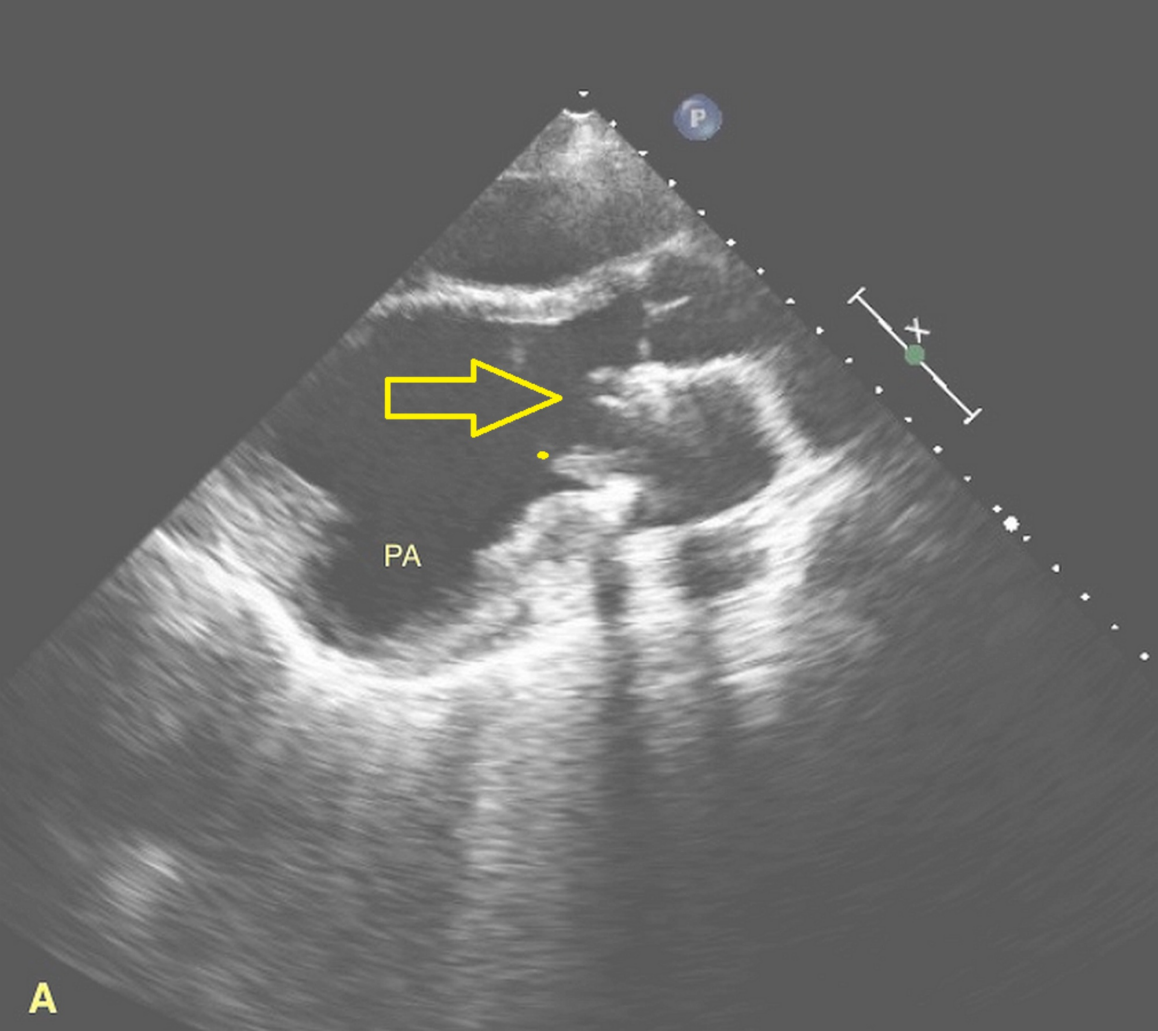
Two-dimensional echocardiography showing the left ventricular pseudoaneurysm (PA).

**Figure 2:**
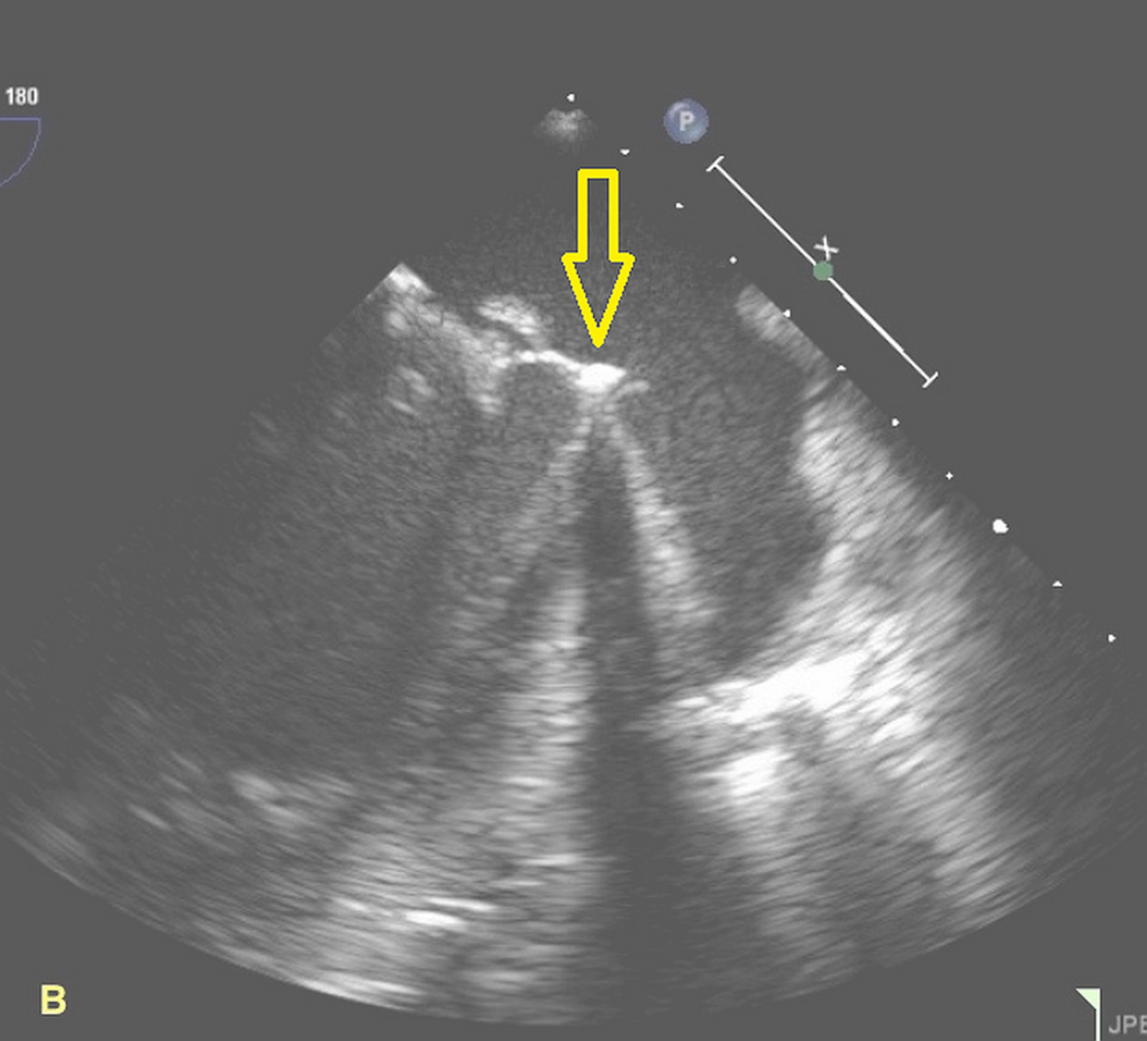
Two-dimensional echocardiography showing the prosthetic mitral valve along with its vegetations.

**Figure 3:**
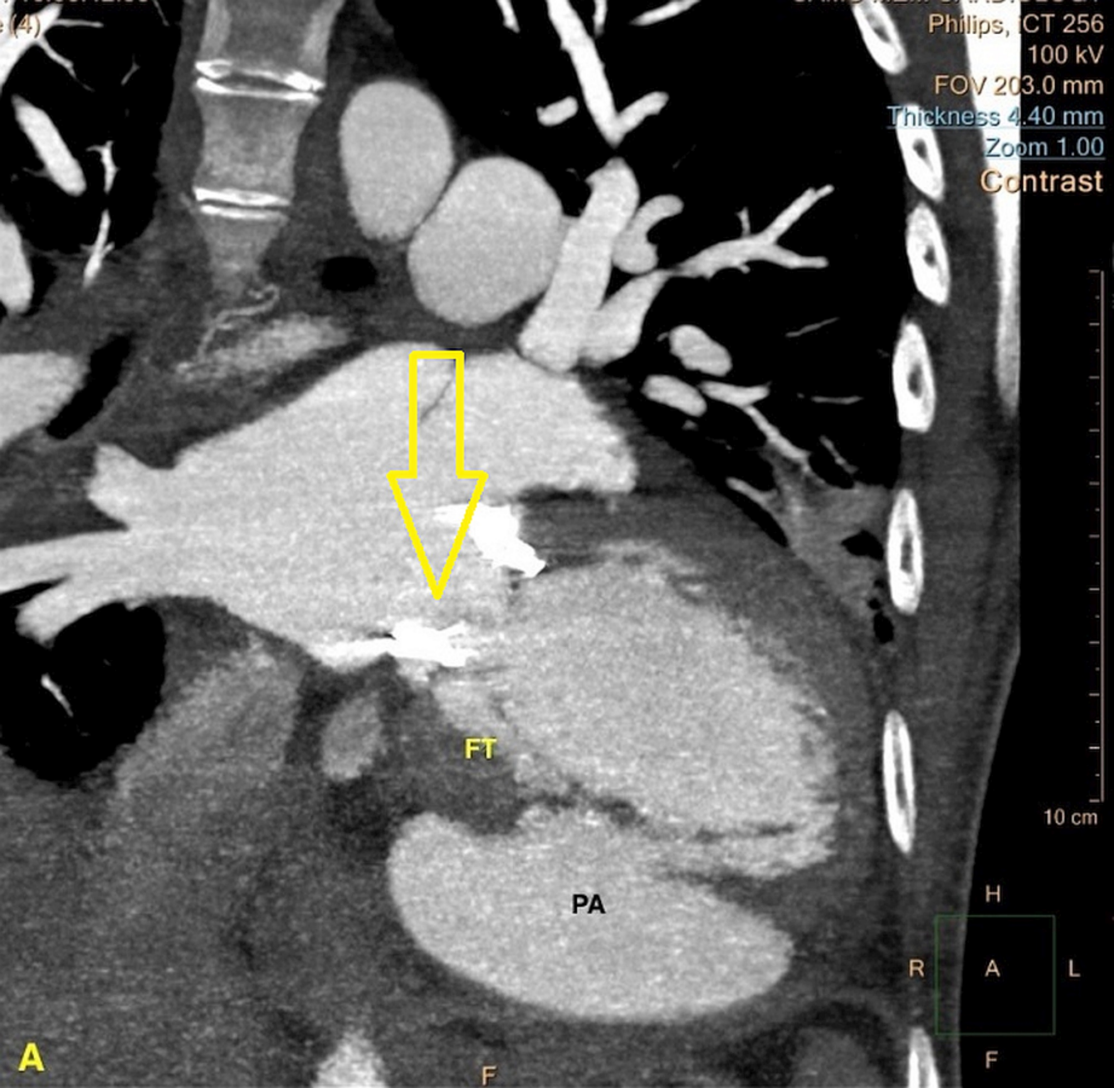
Cardiac computed tomography (cardiac CT) image showing the fistulous tract (FT) communicating from the mitral valve to the left ventricular pseudoaneurysm (PA).

**Figure 4:**
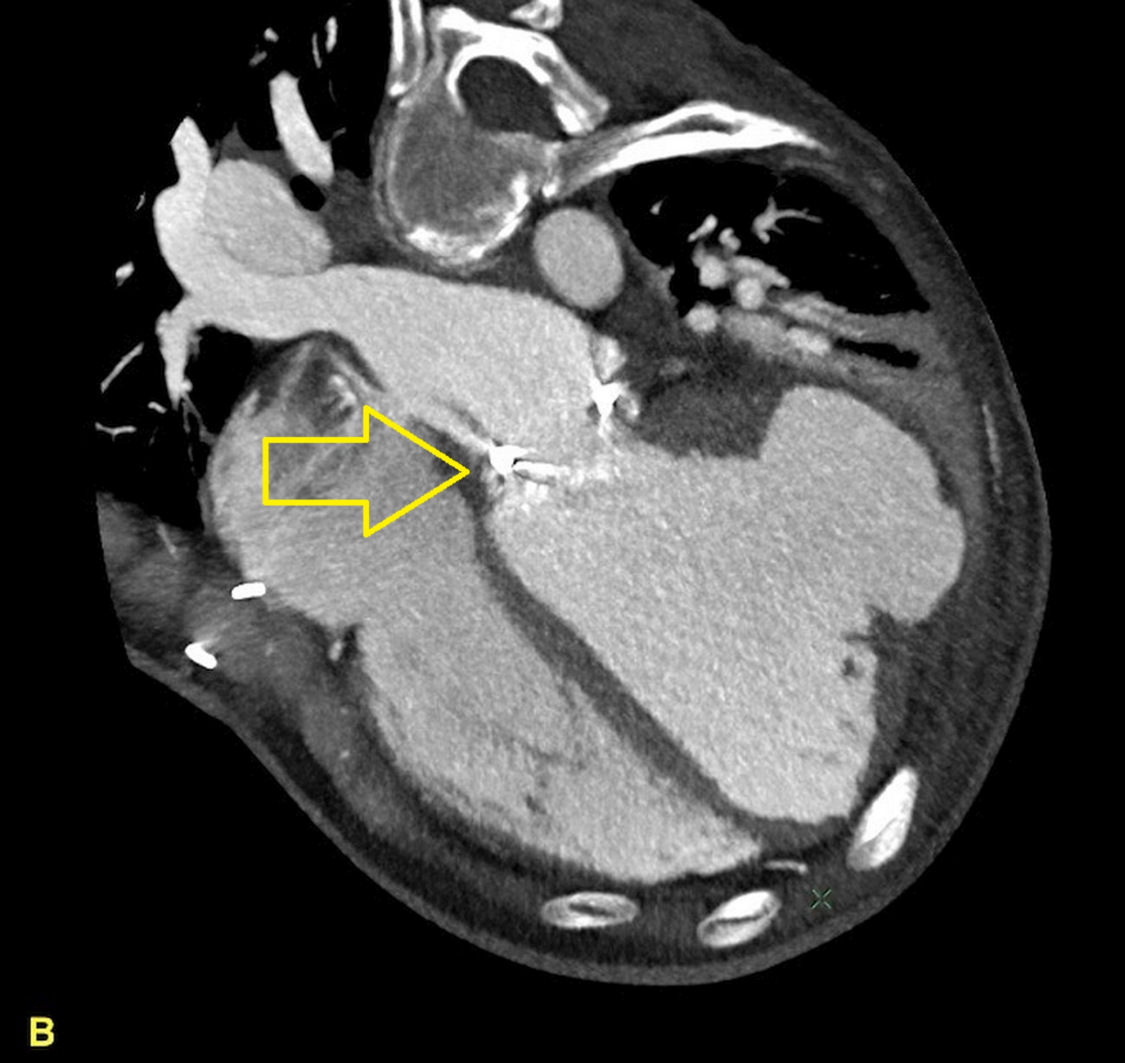
Cardiac computed tomography image showing other cardiac chambers along with the pseudoaneurysm (PA).

A 40-year-old homeless female presented from an outlying facility with chills and body aches for two weeks. Her medical history was significant for hepatitis C, mitral valve (MV) replacement secondary to MV endocarditis, and IV drug use. At presentation, she was in septic shock, and her blood cultures subsequently yielded group A streptococcus. Of note, she stated that her last use of IV drugs was two weeks before presentation. Fluid resuscitation, antibiotics, and vasopressors were appropriately utilized. Transesophageal echocardiography revealed infected mitral valve leaflets, 2-3 cm vegetation in the left atrium, a 0.9 cm atrial septal defect with the left to right shunt, and LV ejection fraction of 15%. No abscesses were visualized. Two weeks later, transthoracic echocardiography revealed a large LVPA originating from the posterolateral wall as shown in Figures 1-2. Cardiac computed tomography (cardiac CT) scan further elucidated the PA that measured 6.9 x 8.4 x 7.2 cm as shown in Figures 3-4.

**Figure 5:**
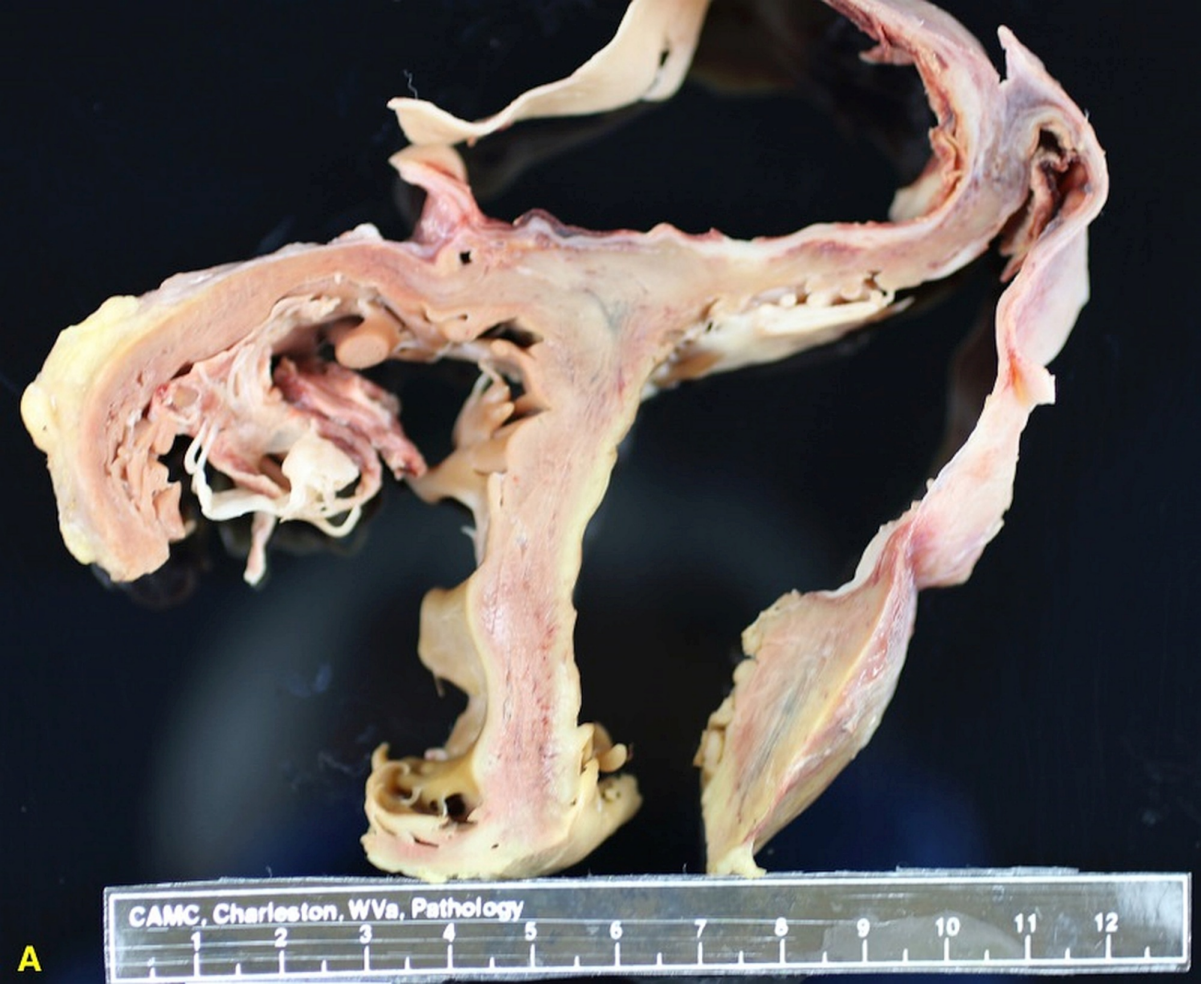
Autopsy image of the heart showing thick left ventricular wall tissue in contrast to the thin pseudoaneurysmal walls – cut and displayed in the upper right-hand corner.

**Figure 6:**
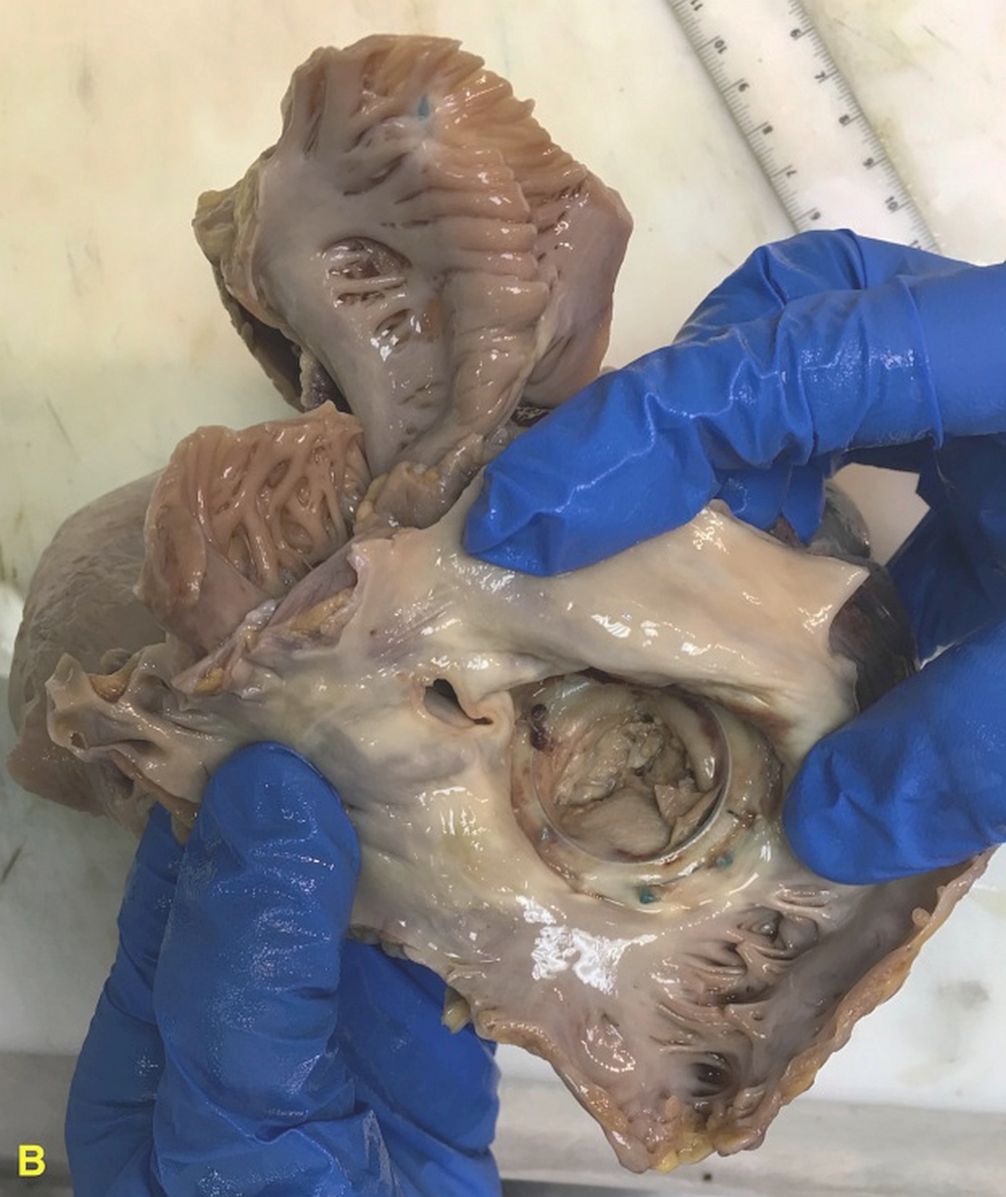
Autopsy image of the heart showing the prosthetic mitral valve.

The cardiac CT also disclosed a fistulous tract communicating from the infected MV annulus to the aneurysmal portion of the LV. A left heart catheterization was performed to rule out a septic embolus to the coronary vessels as a cause for the PA. No coronary artery disease (CAD) or suspicious lesions were discovered. The sheer size of this PA placed this patient at a very high surgical risk. Various institutes were contacted for LVPA repair and redo MV replacement. Due to the high perioperative mortality and poor healthcare coverage, attempts to transfer the patient to tertiary cardiac care centers were futile. The patient’s ongoing drug use also affected her candidacy for MV replacement. When stabilized she was discharged from hospital care on antibiotics and supportive cardiac medications (beta blocker and ACE-inhibitor). She returned to the hospital six weeks later in cardiogenic shock and multi-organ failure. Repeat imaging revealed significantly increased LVPA size. Despite optimal medical management, the patient’s condition deteriorated, comfort care measures ensued, and she ultimately succumbed to her illness. An autopsy revealed cardiomegaly with the heart weighing 580 g. The PA measured 11 x 10 x 5 cm in size and its wall ranged from 0.5 up to 1 cm in thickness as shown in Figures 5-6. No myocardial or aneurysmal ruptures were identified. Microscopic examination of the LVPA tissue showed that the wall was composed primarily of fibrin and organizing thrombosis, with no endocardium or myocardium present, which is consistent with PA.

## Discussion

LVPAs are rare. They have conventionally been portrayed as infrequent complications of MI, particularly of the inferior wall or after cardiac surgery. A single-center retrospective study in China described 10 patients with LVPA between March 2009 and April 2016. MI was the etiology in six cases [4], MV replacement in three cases, and only one case was attributed to suspected endocarditis. This patient had no chest pain, no ST or T-wave changes on EKG, and coronary angiography ruled out CAD or septic emboli. This PA mostly likely developed due to bacterial invasion from the infected prosthetic MV via the fistulous tract, resulting in tissue devitalization and formation of the sac-like outpouching from the LV. True aneurysms are outpouchings that involve full thickness of the LV wall (endocardium, myocardium, and pericardium). In contrast, PAs result from ventricular free wall rupture and are contained only by the pericardium or scar tissue [5]. Also, PAs tend to have narrower necks compared to true aneurysms. Because of these anatomical reasons and the potential for rapid enlargement, PAs are more prone to rupture than true aneurysms and thereby manifesting into surgical emergencies. Most investigators support surgery as the ideal treatment approach for LVPA, although this option is riddled with high mortality rates (23% versus 48% with medical therapy) [6].

## Conclusions

The rise of IV drug use and concomitant bacterial endocarditis within our region is now forcing us to face cardiac complications rarely seen before. PAs, traditionally seen as rare complications of MI or cardiac surgery, are now increasingly emerging with bacterial endocarditis. PAs may result from local bacterial invasion via intracardiac fistulae originating from infected valves. The hospital course of our MV endocarditis patient was complicated by the presence of valvular vegetation, heart failure, and a large PA – all factors associated with higher mortality rates. PAs have no simple solution. Urgent surgical intervention is recommended given the unpredictability of a rupture, even though surgery itself accompanies high mortality rates.
